# Editorial introduction: Biomedicine and life sciences as a challenge to human temporality

**DOI:** 10.1007/s40656-023-00557-8

**Published:** 2023-01-19

**Authors:** Nitzan Rimon-Zarfaty, Mark Schweda

**Affiliations:** 1 Department of Medical Ethics and History of Medicine, University Medical Center Göttingen, Göttingen, Lower Saxony, Germany; 2 Department of Human Resource Management Studies, Sapir Academic College, Hof Ashkelon, Israel; 3 Division of Medical Ethics, Department of Health Services Research, School for Medicine and Health Sciences, University of Oldenburg, Oldenburg, Germany

**Keywords:** Biomedicine, Life Sciences, Temporality, Beginning of life, Middle of life, Later Life

## Abstract

Bringing together scholars from philosophy, bioethics, law, sociology, and anthropology, this topical collection explores how innovations in the field of biomedicine and the life sciences are challenging and transforming traditional understandings of human temporality and of the temporal duration, extension and structure of human life. The contributions aim to expand the theoretical debate by highlighting the significance of time and human temporality in different discourses and practical contexts, and developing concrete, empirically informed, and culturally sensitive perspectives. The collection is structured around three main foci: the beginning of life, the middle of life, and later life. This structure facilitates an in-depth examination of specific technological and biographical contexts and at the same time allows an overarching comparison of relevant similarities and differences between life phases and fields of application.

Recent developments in the fields of medicine, biotechnology, and the life sciences are challenging traditional views of human temporality and seemingly self-evident notions of the temporal extension and structure of life, thus raising novel philosophical, sociological, and bioethical questions and problems. At the same time, new theoretical approaches from existential or hermeneutic philosophy, narrative phenomenology, the social sciences, medical humanities, medical anthropology and science and technology studies (STS) are increasingly acknowledging the significance of time and temporality for understanding contemporary medical practices, biotechnological endeavors, and research paradigms in the life sciences (Arduser & Bennet, 2022; Greco & Graber, 2022; Wahlberg et al., 2021; Martin, 2018; Jain & Kaufman, 2011; Pfleiderer & Rehmann-Sutter, 2006). Our topical collection is aimed to contribute to the systematic exploration and consolidation of this emerging field of research by focusing on the connections and interactions between biomedicine and the life sciences on the one hand and human temporality on the other.

## Background

Traditional images of human existence in time are losing ground. Increasing life expectancies and unprecedented population ageing crack the familiar temporal coordinate system of individual life cycles and intergenerational relations (Kunkel & Settersten, Jr., 2021). Processes of individualization and de-standardization undermine pre-determined biographical pathways and open a wide range of individual options and perspectives (MacMillan, 2005). In this situation, medical, biotechnological and scientific innovations are increasingly challenging common notions of the life course as a fixed sequence of clearly defined stages and trajectories and promise more choice and control at every point in human life.

Interventions at the *beginning of life* like reproductive genetics or preimplantation and prenatal diagnosis highlight the temporal dimensions of prevention and the anticipatory management of future developments (Sänger, 2015; Roberts & Wasserman, 2009). In doing so, they touch upon assumptions about the original formation and essential characteristics of human existence and its evolvement over time. Thus, scientific research and biomedical practices are oriented at underlying models of stages and trajectories of embryonic and foetal development, their specific (dis-)continuities, attributes and potentials, as well as their respective moral significance (Rimon-Zarfaty et al., 2011; Bock von Wülfingen, 2015; Arni, 2015). Cryopreservation technologies (used for freezing gametes, tissues, and embryos) that offer new possibilities for halting and restarting biological processes add further temporal complexities by introducing new forms of “pre-existence”, suspended or latent life (Lemke, 2019, 2021; Hoeyer, 2017; Radin, 2013), thus manipulating developmental continuity over time. Analogous observations can be made with regard to the (bio-)medical treatment of neonates and very young children. New scientific and medical developments and possibilities in this field create new critical points in time for prevention or intervention and thus provoke ideas and arguments regarding open future, irreversible decisions, and prediction of future developments or even personal existence and identity. These aspects become relevant in paediatric practice, for example in the treatment of critically ill neonates (Leuthner, 2014) or intersex children (Meoded Danon, 2014; Feder, 2014), and are often accompanied by ethical discussions of paternalism, proxy decision-making, and anticipated consent (Goold et al., 2019).

In a similar manner, medical interventions in the *middle of life* are often concerned with the irretrievable passing of time. Thus, many reproductive technologies are aimed to expand the time frames of fertility and procreation or even to overcome the limitations of the “biological clock” (Rimon-Zarfaty & Schweda, 2019). Technologies such as IVF (Gershoni & Low, 2021) and in particular social egg freezing appear as medical-technological solutions that can reconcile the tension between the rhythms of “biological time” and the regimes of “social time” (Waldby, 2015; Kroløkke, 2019). They thus raise new issues and controversies about human temporality, including the adequate temporal structure and acceptable biographical timing of reproduction, parenthood, and generational relations (Rimon-Zarfaty & Schweda, 2019; Bühler, 2015). For example, the pertinent discussions bring to the fore the issue of postponing motherhood and the connections between women’s biographies, (gendered) time perceptions and allocations, and reproductive decision making (Leccardi, 2013, 2005; Epstein & Kalleberg, 2004). Some commentators construct reproductive technologies aimed at extending women’s fertility as a deviance from the “natural”, “normal” or “ideal” life course, thus raising questions of “best” or “proper” reproductive timing and the legitimacy of motherhood at advanced ages (for a critical discussion see: Bernstein & Wiesemann, 2014).

Eventually, biomedical and technological interventions focusing on *later life* frequently touch upon aging and the finality of human existence in time. Especially developments in geriatric treatment and end-of-life care, e.g., regarding pharmaceutical therapies or surgical interventions, challenge traditional notions of “medical futility” and therapeutic nihilism in later life and increasingly blur the distinction between “standard” care and “exceptional”, life prolonging measures (Kaufman, 2015). Furthermore, medical and technological developments in intensive care of patients at different levels of coma or even in a state of brain-death, shift the demarcation lines between life and death and thus raise ethical issues of ending treatment and proxy decision-making (Meoded Danon, 2016; Magnus et al., 2015; Kuehlmeyer et al., 2012). Finally, innovations in the fields of preventive or anti-aging medicine contest traditional deficit-oriented views of ageing, the life course, and human finitude. They further promote new ambitious expectations and standards regarding health, functionality, fitness, wellbeing and meaningful prospects for later life (Overall, 2003). The more ambitious branches of biogerontology even inspire “transhumanist” visions of “radical life extension” or “biological immortality” (Rose, 2004) (for a comprehensive interdisciplinary discussion and conceptual analysis of aging and its meaning(s) in light of normative sociocultural perceptions and contemporary scientific practices see also the recent TC of HPLS “Rethinking ageing” (Blasimme et al., 2021)).

All in all, these and similar biomedical and technological innovations challenge common understandings of human temporality and thus raise philosophical as well as empirical questions. They fuel controversial ethical debates about appropriate timing and the individually desirable as well as socially acceptable temporal structure of human life: How is the emergence and development of biomedical technologies and life sciences interwoven with particular understandings and practices regarding human temporality? What is the role of medical diagnoses and therapies as well as technological artefacts in generating new temporal perspectives (Pateraki, 2019; Wajcman, 2019) and even new temporal modes of existence? What does it mean for the ethical evaluation of biomedical practices and life science paradigms that human life is traditionally interpreted as a process with a particular temporal extension and structure, including a sequence of phases or stages connected to different social roles and moral norms and expectations (Schweda, 2017)? What does it mean that certain moral questions and issues in the context of modern biomedicine, technology, and life sciences usually arise at a particular point in the human life course? How do socially based “clocks” or “standard times” (Zerubavel, 1982) interact with the development of medical practice and its understandings? How do notions of temporality change throughout the history of the life sciences, linked to emerging epistemological perspectives and shifting sociocultural regimes (Bock von Wülfingen et al., 2015)? To what extent do they take shape in – and differ between – particular sociocultural contexts (Nowotny, 1992) or gendered perceptions (Leccardi, 2005)?

## Conception, structure, and contributions

These and similar questions provided the original impetus for this topical collection. In the spring of 2019, the editors organized a workshop on biomedicine, biotechnology, and human temporality at the University of Oldenburg. The international interdisciplinary group of contributors included scholars from Germany, Israel, the Netherlands and Switzerland, specializing in the philosophy of science, bioethics and medical ethics, law, sociology, and anthropology. The ongoing discussions and collaborations that originated in this workshop provide the basis and context for the contributions to this collection. The overarching objective is to develop a more concrete, empirically informed, and culturally sensitive perspective regarding time and temporality in the context of biomedicine and the life sciences. The issue is structured around three main foci: the beginning of life, the middle of life, and later life. This enables a systematic overview and comparison of relevant similarities and differences.

### Handling futurity: Health across the lifespan and biomedical interventions at the beginning of life

The opening piece of this topical collection provides an overarching point of view. Combining philosophical and biomedical perspectives, philosopher Ari Schick (Jerusalem) develops an epistemological understanding of health as temporally extended. The contribution starts from an exploration of evolving images of medical practice and emerging temporal aspects of health. Contrasting this perspective with Boorse’s biostatistical theory of health in terms of “normal species functioning” or “absence of disease”, Schick then develops an alternative conception of health that incorporates temporality across the lifespan. By bringing together biomedicine and epidemiology with philosophy of biology and related views on the nature of living organisms, he outlines a process ontology and further establishes a unified "life course process theory of health". Finally, Schick points out implications of this temporal approach to health for different fields of biomedicine that are discussed in more detail in subsequent contributions to this collection, especially reproductive health, healthy ageing, and end-of-life decision-making.

The following contributions focus on specific biomedical and technological issues at the beginning of life. Medical sociologist Limor Meoded Danon (Safed) provides a temporality-sensitive analysis of the treatment of intersex infants, that is, children with variation of sex development (VSD). Based on extensive qualitative social research with medical professionals as well as intersex people and their parents, Meoded Danon identifies three different sociomedical approaches of addressing the uncertainty surrounding intersex/VSD bodies and the related temporal conflicts and complexities: The corrective-concealing approach involves early surgery and hormonal treatments intended to “normalize” patients with intersex bodies/VSD and shape their future soma-sexual development and socialization in order to conceal ambiguity and uncertainty. The preventive approach aims at erasing the past and controlling the future by utilizing selective reproductive technologies to predict, control, and prevent the existence of intersex babies. The wait-and-see approach takes intersex bodies as a natural variant, encourages parents to embrace the social, physical and emotional aspects of uncertainty and live in the present in order to gain time for decision-making. The author finally applies the lenses of biopolitics and phenomenology to conceptualize temporality as a prominent sociopolitical agent in controlling and managing human bodies and related uncertainties.

Jozef H. H. M. Dorscheidt’s (Groningen) contribution then turns to a highly sensitive field of biomedicine connecting the beginning and the end of human life: end-of-life decision-making in the context of pediatric care. In his paper, Dorscheidt describes current legal regulations and medical practices in two European countries: the Netherlands and Belgium. Both have adopted regulations that permit physician-assisted suicide and euthanasia in minors who experience hopeless and unbearable suffering under the condition that the minor involved is legally competent and able to express an authentic and lasting wish to die. The contribution represents a highly original legal approach as it highlights temporality and its legal significance for end-of-life decision making in pediatrics and discusses legal lessons to be learned from a time-sensitive perspective. Particular attention is paid to the question of whether incurable and suffering minor patients have, from a legal point of view, a sufficient ability to choose death and to the way temporality affects competency and its assessment. These considerations are further connected to notions of the continuity of personal development. The paper finally discusses whether such notions can be (made) productive for assessing minor patients’ end-of-life requests.

### Mastering the passing of time: Reproductive technologies and enhancement in the middle of life

The second section of this topical collection focuses on the middle of life. The section presents different aspects and examples of the usage of scientific advancements and medical technologies as a means of controlling and mastering biological and biographical temporality. It opens with a contribution by anthropologist Nolwenn Bühler (Lausanne) that focuses on the connection between reproductive biomedicine, the challenges of reproductive timing, and the age limits of motherhood. The piece draws attention to the transgressive potential of reproductive technologies as they blur the biological landmark of menopause – governing and organizing reproductive lives. Bühler employs the concept of “biotemporality” in order to analyze the ways such technologies challenge the notion of maternal age and its ontological foundations. Relating to relevant literature dealing with reproductive technologies and reproduction, she further discusses the reconfiguration of the ontological boundaries of the facts of life. Based on anthropological research on reproductive biomedicine (i.e., egg donation and social egg freezing) and age-related infertility in Switzerland, the piece uncovers the views, attitudes and negotiations of medical experts regarding the age limits of motherhood, elucidating the binaries of the normal/pathological and the biological/social. By that, the author provides a contemporary example of different configurations of moral reasoning, highlighting a change in the understanding of what is natural and reflecting new negotiations of the normative order.

Sociologist Nitzan Rimon-Zarfaty (Göttingen and Hof Ashkelon) and bioethicist Silke Schicktanz (Göttingen) combine historical, theoretical, and socio-empirical insights for a temporal analysis of contemporary practices of cryo-fertility. The piece starts with a broad historical overview of cryo-fertility and its developments. It then applies a theoretical framework examining how cryobiology and cryo-technologies in the wider sense open up a new epistemic perspective interconnecting biology and temporality while creating novel forms of “latent” or “suspended” lives. In what follows, the authors focus on social egg freezing (SEF) as a particular field of application of cryo-fertility and present findings from a cross-cultural comparison of the views and experiences of German and Israeli SEF users. The case study reveals three different types of “reproductive temporalities”: postponing motherhood/reproductive decisions (German users); singlehood and “waiting” for a partner (Israeli and German users); and the planning of and hope for multiple children (Israeli users). The authors further discuss these temporalities in terms of the “extended present”, “waiting”, and “reproductive futurism”. Emphasizing the relevance and importance of emerging gendered and cultural imaginaries, they finally draw conclusions for the theoretical framework of cryopolitics.

In the final contribution of this section, philosopher and medical ethicist Claudia Bozzaro (Kiel) presents a critical analysis of a variety of emerging medical enhancement technologies aimed at transcending the natural temporal rhythms and limitations of human life. To this end, Bozzaro reflects upon the implications of the finitude and fugacity of individual lifetime for leading a good life. Combining philosophical and sociological perspectives, she first analyses the increasingly negative experience of the “passing of time”, in particular in the context of contemporary individualized, capitalist, and consumerist societies. Against this backdrop, she then critically examines the usage of three biomedical enhancement technologies representing recent developments in the life sciences: social egg freezing, anti-aging medicine, and physical-/neuro-enhancement, as well as their attempts at controlling or halting the passing of time or expanding the lifespan. Embracing an existentialist perspective, Bozzaro arrives at the conclusion that such attempts are doomed to fail because the awareness of time’s passing is a necessary precondition for leading a good life.

### Projecting finality: Imagining and planning later life

The final section of this collection turns to temporal aspects in the context of ageing, old age, and the end-of-life. The contribution of sociologist and bioethicist Julia Perry (Göttingen) combines both theoretical and empirical reflections on advance care planning and advance research directives (ARDs) in the context of dementia. The author first outlines the theoretical concept of anticipation and its key dimensions as discussed in the sociology of the future. She then provides an overview of dementia, advance planning in medical and research contexts, and the current practice of research involving people with decreased decisional capacities. This section presents an interpretation of advance planning as a form of anticipation whose epistemic, social, and normative challenges or implications need further examination. Against this backdrop, the contribution offers insights from qualitative empirical research assessing ARDs for people with dementia or cognitive impairment in Germany and devises a framework for anticipatory decision-making taking into account perceptions of time and liminality entangled with notions of uncertainty and changing values, needs, and preferences in the dementia trajectory.

In the collection’s closing contribution, philosopher and medical ethicist Mark Schweda (Oldenburg) and bioethicist Karin Jongsma (Utrecht) criticize the cultural metaphor of dementia as a “death while alive”. By implementing a historical perspective, the authors first trace the development and the philosophical premises of this image. Against this backdrop, the piece undertakes a critical inspection of the implications and consequences of this metaphor in terms of scientific understandings as well as social attitudes and behaviors regarding people with dementia. Employing a life course perspective that considers the ethical significance of the temporal extension and structure of human life, the authors problematize the way the “death while alive” metaphor identifies dementia with a deviation from the biographical norm and the normative standards of age-appropriate behavior, and thus as a disruption in an assumed temporal order of existence. They draw conclusions with regard to medical and nursing ethical debates in the context of dementia, e.g., concerning self-determination, surrogate decision making, and advance directives, and stress the theoretical importance of a biography- and culture-sensitive approach to philosophical and ethical reasoning in the context of the life sciences.

Overall, this topical collection thus offers an exploratory overview of the manifold interconnections between ongoing developments in medicine, biotechnology, and the life sciences on the one hand, and perspectives of human temporality, the life course, and intergenerational relations on the other. It thus sheds light on the variety of mutual relations and interactions in which medical, biotechnological, or scientific innovations either challenge or transform human temporality – or are themselves perceived, negotiated, and implemented based on social ideas, moral standards, as well as gendered and cultural imaginaries of temporality. In doing so, the collection highlights pertinent temporal concepts and attitudes that deserve closer interdisciplinary examination, including continuity and disruption, letting time pass or halting (“freezing”) it, preceding, postponing, waiting, planning, anticipating, and ending. In addition, the different contributions also offer a useful contextualization with regard to particular medical, biotechnological, and scientific settings, as well as different stages of individual development and generational cycles. The collection thus connects motivations behind the usage of medical and biotechnological innovations, the ways they are embedded in everyday life and different sociocultural contexts, the power relations and biopolitical mechanisms involved, and the relevant ethical concerns and philosophical debates.

## Data Availability

Not applicable.
