# Correction to: Editorial introduction: Biomedicine and life sciences as a challenge to human temporality

**DOI:** 10.1007/s40656-023-00565-8

**Published:** 2023-02-28

**Authors:** Nitzan Rimon-Zarfaty, Mark Schweda

**Affiliations:** 1 Department of Medical Ethics and History of Medicine, University Medical Center Göttingen, Göttingen, Lower Saxony, Germany; 2 Department of Human Resource Management Studies, Sapir Academic College, Hof Ashkelon, Israel; 3 Division of Medical Ethics, Department of Health Services Research, School for Medicine and Health Sciences, University of Oldenburg, Oldenburg, Germany

**Correction to: History and Philosophy of the Life Sciences (2023) 45:3**

10.1007/s40656-023-00557-8

In this article the author name Nitzan Rimon-Zarfaty was incorrectly written as Nitzan Rimon-Zarfay.

The original article has been corrected.

